# Validation of an mHealth App for Depression Screening and Monitoring (Psychologist in a Pocket): Correlational Study and Concurrence Analysis

**DOI:** 10.2196/12051

**Published:** 2019-09-16

**Authors:** Roann Munoz Ramos, Paula Glenda Ferrer Cheng, Stephan Michael Jonas

**Affiliations:** 1 Department of Medical Informatics RWTH Aachen University Hospital Aachen Germany; 2 College of Education Graduate Studies De La Salle University-Dasmarinas Dasmarinas City, Cavite Philippines; 3 Vivech System Solutions Inc Manila Philippines; 4 Department of Psychology College of Science University of Santo Tomas Manila Philippines; 5 Department of Informatics Technical University of Münich Münich Germany

**Keywords:** mobile health, depression, validation, Psychologist in a Pocket, PiaP

## Abstract

**Background:**

Mobile health (mHealth) is a fast-growing professional sector. As of 2016, there were more than 259,000 mHealth apps available internationally. Although mHealth apps are growing in acceptance, relatively little attention and limited efforts have been invested to establish their scientific integrity through statistical validation. This paper presents the external validation of Psychologist in a Pocket (PiaP), an Android-based mental mHealth app which supports traditional approaches in depression screening and monitoring through the analysis of electronic text inputs in communication apps.

**Objective:**

The main objectives of the study were (1) to externally validate the construct of the depression lexicon of PiaP with standardized psychological paper-and-pencil tools and (2) to determine the comparability of PiaP, a new depression measure, with a psychological gold standard in identifying depression.

**Methods:**

College participants downloaded PiaP for a 2-week administration. Afterward, they were asked to complete 4 psychological depression instruments. Furthermore, 1-week and 2-week PiaP total scores (PTS) were correlated with (1) Beck Depression Index (BDI)-II and Center for Epidemiological Studies–Depression (CES-D) Scale for congruent construct validation, (2) Affect Balance Scale (ABS)–Negative Affect for convergent construct validation, and (3) Satisfaction With Life Scale (SWLS) and ABS–Positive Affect for divergent construct validation. In addition, concordance analysis between PiaP and BDI-II was performed.

**Results:**

On the basis of the Pearson product-moment correlation, significant positive correlations exist between (1) 1-week PTS and CES-D Scale, (2) 2-week PTS and BDI-II, and (3) PiaP 2-week PTS and SWLS. Concordance analysis (Bland-Altman plot and analysis) suggested that PiaP’s approach to depression screening is comparable with the gold standard (BDI-II).

**Conclusions:**

The evaluation of mental health has historically relied on subjective measurements. With the integration of novel approaches using mobile technology (and, by extension, mHealth apps) in mental health care, the validation process becomes more compelling to ensure their accuracy and credibility. This study suggests that PiaP’s approach to depression screening by analyzing electronic data is comparable with traditional and well-established depression instruments and can be used to augment the process of measuring depression symptoms.

## Introduction

### Background

Mobile technology has gained widespread acceptance and is seamlessly integrated in day-to-day activities, expanding especially into the field of health care. Mobile health (mHealth) is considered to be among the fastest growing sectors nowadays with a compound annual growth rate of 32.5% [1] and more than 259,000 apps available from over 59,000 publishers worldwide. Although mHealth apps definitely have their inherent appeal and value, very little attention and effort has been given to establish their scientific integrity or validity [2-4]. This is especially true in apps targeting mental health.

Validity ensures whether a novel approach is comparable with or is in agreement with the existing traditional methodology or instrument. Current scientific status of apps targeting mental health and behavioral disorders lack supporting data and empirical evidence on efficacy and outcome. Overall, studies on app validation and clinical effectiveness have not kept up with the pace of app development [5]. For instance, a scant 2% or 32 out of the 1536 downloadable mHealth apps for depression in 2013 were based on scientific publications [6]. Only 14 of 1065 articles on smartphone apps for bipolar and major depressive disorders reported having conducted scientific studies, mostly pilot or feasibility tests [7]. The United Kingdom’s National Health Service has a list of 14 recommended apps in their library, 4 of which provide evidence based on patient reports [8].

In addition to the general lack of science-based development, most existing research on mobile technology and mental health care is methodologically limited with very small sample sizes [9,10] or are supported with feasibility studies only [11,12]. This shows the need for validation of accuracy and reliability of published apps.

The challenge of the validation process is the absence of a universal agreement on mHealth app metrics to identify high quality mobile apps, such as standardized evaluation and rating tools. Setting common evaluation benchmarks for existing health apps can be a challenging task because of their varied features, functions, and suitability. Although rating scales and classification platforms have been developed for mobile apps [4,13], these criteria cannot be implemented to all mHealth apps. Even major professional organizations, such as the American Psychological Association and the American Psychiatric Association, have yet to provide general guidelines as basis for mobile app evaluation [14]. The US Food and Drug Administration does not intend to regulate apps that appear to be of low risk nor transform a smartphone into a medical device [15].

### Objective

This paper tackles the issue of mHealth app credibility by applying the psychometric approach of construct validation to a mobile app in mental health. Validation aims to determine whether or not relationships with other variables exist, and, if such relationships exist, to what magnitude. In this work, we focused on the validation of an app in depression detection through ecological momentary assessment (EMA).

EMA allows for a continuous detection of an individual’s subtle and incremental mood changes during daily life. Compared with traditional psychological assessments such as self-reports and questionnaires, EMA’s feature of real-time assessment avoids or reduces recall bias through recurrent and repeated data recording of daily cognitive and emotional dynamics. Various studies suggest that EMA provides accurate data regarding depression symptoms [16]. Mobile apps can support EMA through unobtrusive monitoring of day-to-day activities and social interactions.

The *Psychologist in a Pocket* (PiaP) [17] is an Android-based mental health app which aims to support and assist mental health professionals and complement traditional assessment approaches in depression detection and monitoring through EMA [18]. As it relies on EMA, PiaP reduces or eliminates the limitations of retrospective measurements (patient interviews and self-report) currently being used in mental health care assessment. Examples of the limitations that PiaP addresses are the reliance on the patient’s memory and the overlooking of subtle or underreported symptoms by mental health practitioners.

PiaP’s basic assumptions are as follows: (1) Everyday language—its usage, content, and themes—is a reliable indicator of the state of one’s mental health; (2) Individuals tend to reveal personal information when using electronic media; and 3) Depressed or depression-prone individuals tend to self-focus and to ruminate on the negative aspects of their lives. PiaP aims at detecting changes in the nature of electronic text inputs through a lexicon of words in English and Tagalog related to depression, which were developed using both top-down and bottom-up processes (see [19] for app details and [18] for technical details). Sources for the lexicon were (1) symptom classification systems of the Diagnostic and Statistical Manual of Mental Disorders (DSM)-5 criteria for major depressive disorder and the International Statistical Classification of Diseases and Related Health Problems–10 criteria for depressive disorder, (2) focus group discussions, (3) interviews with mental health professionals, and (4) established psychological tests. As a result of these approaches, PiaP lexicon has a total of 13 symptom categories: mood, interest, appetite and weight, sleep, psychomotor agitation, psychomotor retardation, fatigue, guilt and self-esteem, concentration, suicide, alcohol and substance abuse, anxiety, and histrionic behavior. In addition, PiaP includes the category of first-person pronouns to reflect self-focus tendencies.

In the following sections, the construct validation of the PiaP depression lexicon is described. We hypothesize (Hypothesis 1, H1) that construct validity of the PiaP can be proven based on the measures for (H1.1) congruent, (H1.2) convergent, and (H1.3) divergent construct validations. In addition (Hypothesis 2, H2), statistical agreement of the PiaP with a test measuring the same variable (Beck Depression Index [BDI]-II) is hypothesized.

## Methods

### Tripartite Model of Test Construction

The development and validation of the PiaP lexicon is based on the tripartite model of test construction [20,21]. PiaP lexicon progressed through 3 stages, which are (1) theoretical-substantive (test items are generated according to theoretical requirements), (2) internal-structural (rational items are subjected to validation to establish internal consistency via construct validation, item analysis, and tests), and (3) external-criterion (entire test is investigated for its measurement of its construct as compared with other established measurement tools). A major advantage of this model is that it combines the strength of each phase in coming up with a reliable and valid measurement tool [22]. Items that are deemed to be inadequate are removed throughout the phases.

As PiaP is designed for depression-screening purposes, it underwent the technical phases of item or keyword construction. As a result, 2 versions (V1 and V2) of the PiaP lexicon were developed for validation. Stage 1 of the tripartite model provided the PiaP V1 keywords. Included are main keywords, derivatives of main keywords, and spelling variations (PiaP V1 total=835,286). During stage 2, PiaP V1 underwent internal validation to determine its internal psychometric properties (content validity, item analysis, and internal consistency). Only internally valid depressive-symptom keywords from PiaP V1 were included in PiaP V2 for use in stage 3 (external validation; PiaP V2 total=781,936).

Research proposal was first subjected to ethical review and approval by the Ethics Review Committee of the Graduate School, University of Santo Tomas (Manila, Philippines). After obtaining ethics approval, several potential universities were considered. Research letters were sent out to 6 universities in Manila and nearby provinces. Of the 6, 3 universities agreed to take part in the 3-stage study.

In this paper, only the results from stage 3 of the tripartite model are presented and discussed (see [19] for stages 1 and 2).

### Participants

A total of 510 college students from stage 2 initially agreed to participate for 2 weeks during stage 3 of the research. Using homogenous sampling, they were purposively selected from Metro Manila colleges and universities, based on the following selection criteria: (1) must be enrolled in a tertiary academic institution at the time of data gathering, (2) should be aged between 16 and 25 years, (3) should have a mobile device that functions under Android operating system for PiaP to function, and (4) should have internet access at the time of PiaP download and upload of their encrypted data to the researcher. (Please see Multimedia Appendices 1 and 2 for sample screenshots; the presentation for the app is available in Multimedia Appendix 3).

Of the 510 participants, 332 could not be contacted immediately after inclusion despite follow-ups and reminders; thus, they were considered as *immediate dropouts*. After a 2-week administration of the PiaP V2, the remaining 178 participants were required to complete the following psychological tests to prove the research hypotheses: (1) Beck Depression Inventory (BDI)-II (H1.1 and H2); (2) Center for Epidemiological Studies–Depression (CES-D) Scale (H1.1); (3) Affect Balance Scale (ABS)–Negative Affect (H1.2); (4) Satisfaction With Life Scale (SWLS; H1.3); and (5) ABS–Positive Affect (H1.3).

Only 53 completed both the trial period and data collection. Participants (n=125) were excluded from data analysis for the following reasons:

- Sent empty encrypted psychological test files (n=2)
- Did not send encrypted psychological test files for unknown reasons (n=3)
- Did not send encrypted psychological test files because of internet problems (n=3)
- No data recorded owing to not following PiaP V2 setup instructions (n=4)
- Had changed phones (from Android to iPhone; n=5)
- Had Android version incompatibility with PiaP V2 (n=6)
- Dropped out (n=10)
- Experienced unexpected technical difficulties (n=10)
- Did not accomplish all psychological tests (n=33)
- Discontinued app after using PiaP V2 for a couple of hours/few days (n=49)

Data collection and analysis was based on 53 undergraduate students with a mean age of 17.42 (SD 1.03) years (Table 1). The average BDI-II score is 17.49 (SD 11.15), which is equivalent to a mild level of depressive symptoms.

### Ethical Considerations

Voluntary participation was emphasized. Informed consent forms were distributed and filled up during each of the research stages. Moreover, participants were duly informed and reminded of the right to withdraw from the study at any time.

As privacy, data security, and anonymity of respondents were of paramount importance, several points were ensured:

1. Downloading the app needs only 1-time internet access. After downloading, PiaP runs offline. As a result, each of the participant’s text inputs were stored locally (ie, in their mobile devices).
2. Only the researchers have sole and exclusive access to participant data (password protection). Participants were instructed to upload encrypted files to a designated cloud-based storage using the PiaP app. After data collection, all data were deleted or removed from the cloud storage.
3. In lieu of names, each participant was assigned and identified via a number code.

In addition, participants who were found to have significant BDI-II depressive symptom scores that warrant attention were individually referred to a clinical psychologist or counselor from their respective universities.

**Table 1:** Participant statistics (N=53).

Characteristics		Value
Gender (female), n (%)		43 (81)
Age (years), mean (SD)		17 (1)
Number of years at university, mean (SD)		2 (1)
BDI^a^-II score, mean (SD)		18 (12)
**BDI-II level, n (%)**		
	Minimal	21 (40)
	Mild	13 (24)
	Moderate	7 (13)
	Severe	12 (23)

^a^BDI: Beck Depression Inventory.

### Construct Validation Process

In psychometrics, one type of validity is construct validity—the extent to which a measure adequately assesses the construct it purports to assess [23]. A *construct* (also known as *psychological construct*) is an attribute measured in a test. As a construct is generally not directly observable, this is validated through evidences of its relationships or correlations with psychometrically sound psychological tests, which either measure the same attribute or a different construct.

To accomplish this, 3 types of construct validity can be analyzed: (1) *Congruent construct validity* refers to a test’s congruency or relationship with a known valid and reliable measure of the same construct [24] (eg, 2 measures of depressive symptoms); (2) *Convergent construct validity* correlates scores on a new test with the scores of established tests of related constructs [25] (eg, negative affect and depressive symptoms); and (3) *Divergent construct validity* provides discriminant evidence by proving that a particular test has low correlations with measures of unrelated constructs [26] (eg, life satisfaction and depressive symptoms).

To prove hypotheses H1.1, H1.2, and H1.3, the congruent, convergent, and divergent constructs needed to be selected.

The study’s construct is *depressive symptoms*. It is characterized by negatively valenced words (words that describe unpleasant emotions) grouped according to 1 of the PiaP 13 symptoms based on a prior-developed lexicon and the frequency of first-person pronoun usage (see Cheng, et al [19] and Ramos, Cheng et al [27] for the development of the mentioned lexicon).

For congruent validity, the study characterization is compared with standardized tests for the same construct.

For convergent validity, the construct *negative affect* was chosen as previous researches have indicated a relationship between depression and negative affect [28]. Increases in negative affect, in response to everyday life challenges, reflect vulnerability to depression [29].

For divergent validity, the constructs *positive affect* and *life satisfaction* were chosen. As life satisfaction has been shown to be inversely associated with depression [30,31], positive affect and life satisfaction are considered to be a major indicator of subjective well-being [32]. For the convergent construct, *negative affect* was selected. *Positive affect*, similar to negative affect, is the emotional, affective component of subjective well-being. However, unlike negative affect, positive affect is the *pleasurable engagement with the environment* [33] and can be a protective factor against depression [34]. *Life satisfaction* is a distinct attribute as it constitutes the cognitive component of subjective well-being. It is an overall assessment about one’s current life situation based on his or her personal criteria [32,35,36]. It is highly unlikely that a person who is satisfied with life can also be depressed at the same time [37].

Next, correlation was calculated to determine construct validity of PiaP (*depressive symptoms*) against the following psychological measures:

- Congruent construct validity (H1.1)
  - (1) BDI-II
  - (2) CES-D Scale
- Convergent construct validity (H1.2)
  - (3) ABS–Negative Affect component
- Divergent construct validity (H1.3)
  - (4) SWLS
  - (5) ABS–Positive Affect component

Note that BDI-II and CES-D Scale measure depressive symptoms before testing. Therefore, the PiaP total scores (PTS) of each respondent spanning 2 weeks and 1 week were correlated with BDI-II and CES-D Scale, respectively.

### Statistical Analysis

In determining the construct validity of PiaP against the psychological measures used in the study, Pearson product-moment correlation (PPMC) of scores on all tests were calculated [38]. PPMC was employed to determine the strength of association between PiaP’s interval scales scores with each of the psychological tests. In this research, positive correlations are evidences of congruent and convergent validities, whereas negative correlations are expected in divergent construct validation.

Study findings are explained according to Hinkle et al’s [39] rule of thumb in interpreting the size of the correlation coefficient (Table 2).

To determine the practical significance of the results, Cohen *d* effect size (ES) was used to interpret the correlation values (Table 3). ES presents the magnitude of reported effects in a standardized manner, regardless of the scale used to measure a variable [40].

Although correlation quantifies the degree of relation, it does not automatically imply good agreement between 2 methods. Thus, to prove H2, further statistical validation to compare 2 different types of measurements (PiaP and BDI-II) of the same variable (depression symptoms) was performed by applying Bland-Altman (B-A) plot and analysis. The researchers selected BDI-II as the established psychological test with which PiaP was compared, as this test is considered the gold standard of self-rating scales designed to measure the current severity of depressive symptoms [41].

### Psychologist in a Pocket Normative Structure

PiaP’s set of norms was based on data collected from 924 days of PiaP usage of 510 randomly selected college student participants from the study’s stage 2. Participants’ average number of days of PiaP usage is 10.62. The overall tally of text inputs per day of all relevant words (regardless of symptom category) detected by the depression lexicon is referred to as the PiaP total score (PTS). Specifically, the PTS is increased by 1 score point for each typed word present in the PiaP lexicon. During the 2-week period, a total of 31,336 text inputs from all the participants was obtained, with an average of 11.40 (SD 17.77) text inputs per daily evaluation, with a score range of 0 (no depression-related keyword detected in text inputs) to 164 (maximum number of text inputs detected as matching the keywords in the depression lexicon).

For the interpretation of the PTS, quartiles were calculated to determine the levels of depressive symptoms from normal to critical (Table 4). The normal level represents scores from individuals who do not experience depression yet had typed words representative of depression and its symptoms (eg, for research purposes). Score ranges from above normal to critical levels signify that the text inputs suggest varying degrees of depression as detected by the lexicon.

It is important to note that gender-specific norms were not created as studies with adolescents conclude that gender does not influence depressive symptomatology [42,43].

### Psychological Tests

#### Beck Depression Inventory–II

BDI–II [44,45] is a 21-item self-report measuring the intensity of current depressive symptoms (sadness, pessimism, loss of pleasure, etc) based on the DSM, particularly for ages 13 to 80 years. Respondents report each symptom on a 4-point Likert scale retrospectively for the 2 weeks prior the test. The highest possible score is 63 with minimal (0-13), mild (14-19), moderate (20-28), and severe (29-63) ranges.

#### Center for Epidemiological Studies–Depression Scale

The CES-D Scale, initially developed for epidemiological research, is a 20-item screening tool to detect current depressive symptoms during the week before taking the test, with an emphasis on depressed mood [46,47]. It covers 4 factors: depressive affect, somatic symptoms, positive affect, and interpersonal relations. Respondents choose on a 4-point Likert scale. Scores of 16 and above indicate significant symptoms, with 60 as the highest possible score.

#### Affect Balance Scale

ABS [48] targets objective well-being through the assessment of positive and negative affect. The 10-item scale focuses on feelings experienced by respondents over the past few weeks, with 5 items each to describe positive and negative affect. Respondents choose on a binary scale *Yes* (score of 1) or *No* (score of 0). Total affect balance score is computed by subtracting the negative affect score from the positive affect score and then adding a constant of 5 to avoid values below 0. A score of 0 means low affect balance, whereas 10 reflects high affect balance.

**Table 2:** Interpreting correlation values.

Absolute size of correlation	Interpretation
0.90 to 1.00	Very high positive (negative) correlation
0.70 to 0.90	High positive (negative) correlation
0.50 to 0.70	Moderate positive (negative) correlation
0.30 to 0.50	Low positive (negative) correlation
0.00 to 0.30	Negligible correlation

**Table 3:** Interpretation of Cohen *d* (effect size).

Effect size	Interpretation
0.50	Large
0.30	Medium
0.10	Small

**Table 4:** Psychologist in a Pocket total score interpretation.

Level	Brief description	Psychologist in a Pocket total score range (text input)
Normal	Typical or average number of depression-related keywords typed by an individual without depression	0-19
Above normal	Higher than average amount of depression-related keywords typed by an individual with some (mild) signs of depression	20-38
High	Considerable amount of depression-related text inputs by an individual with possible moderate signs of depression	39-65
Critical	Elevated amount of depression-related text inputs by an individual with a possible clinical or serious case of depression	66-164

#### Satisfaction With Life Scale

The SWLS is designed to measure life satisfaction as a whole and does not tap positive or negative affect, happiness, or satisfaction related to various life domains [49]. Participants indicate how much they agree or disagree with each of the 5 items measuring global satisfaction using a 7-point scale. Participants within the higher score range of 30 to 35 consider life as enjoyable and that major domains of life are well. Scores between 5 to 9 reflect extreme dissatisfaction in multiple areas of life.

## Results

### Descriptive Statistics

In Table 5, we present an overview of the measures used in this study. The number of observations for PiaP reflect the 1-week and 2-week tallies of depression-related keywords (relevant inputted keywords) of the 53 participants as identified by the PiaP depression lexicon. As CES-D Scale is covering only 1 week, it was correlated with the 1-week period, whereas data from the 2-week period was used to correlate with BDI-II scores. There was a notable decrease of depression-related keywords in the second week of PiaP administration.

Depression levels of the participants range from mild to moderate, as indicated by their mean scores in the 2 depression measures used, BDI-II and CES-D Scale. Score in ABS, which comprises ABS–Positive Affect and ABS–Negative Affect, reflect an average level of happiness (ABS total score=5.66). However, for the purposes of this research, we looked at these 2 scale components separately. Participants reported having mild negative affect while experiencing moderate positive affect. Finally, participants are slightly satisfied with their lives, as inferred from the SWLS mean score.

### Hypothesis 1: Construct Validity Correlations

Table 6 presents the correlation coefficient results for the 3 construct validation approaches of 1-week and 2-week PTS with each of the psychological instruments used.

The exact *P* values have been provided below.

**Table 5:** Descriptive statistics (Psychologist in a Pocket and psychological tests).

Measure (score range)	Number of observations	Mean (SD)	Interpretation
PiaP^a^ 1-week (0-3154)	3154 keywords	59.64 (78.238)	High
PiaP 2-weeks (0-5214)	5214 keywords	101.06 (93.140)	Critical
BDI^b^-II (0-63)	53 participants	17.49 (11.154)	Mild
CES-D Scale^c^ (0-60)	53 participants	19.81 (10.958)	Moderate
ABS^d^–Negative Affect (0-5)	53 participants	2.49 (1.589)	Mild
ABS–Positive Affect (0-5)	53 participants	3.15 (1.199)	Moderate
SWLS^e^ (5-35)	53 participants	20.58 (5.716)	Average

^a^PiaP: Psychologist in a Pocket.

^b^BDI: Beck Depression Index.

^c^CES-D Scale: Center for Epidemiological Studies–Depression Scale.

^d^ABS: Affect Balance Scale.

^e^SWLS: Satisfaction With Life Scale.

**Table 6:** Construct validation results (correlation coefficient) and hypothesis (N=53 for all analyses).

Psychological tests	Psychologist in a Pocket, correlation coefficient		Effect size	Hypothesis	Hypothesis support
	1-week	2-week			
BDI^a^-II	—^b^	0.50^c^	Large	Hypothesis 1.1	Yes
CES-D Scale^d^	0.42^c^	—	Medium	Hypothesis 1.1	Yes
ABS^e^–Negative Affect	0.25	0.19	N/A^f^	Hypothesis 1.2	No
ABS–Positive Affect	−0.29^g^	−0.20	Medium	Hypothesis 1.3	Yes
SWLS^h^	−0.29^g^	−0.32^g^	Medium	Hypothesis 1.3	Yes

^a^BDI: Beck Depression Index.

^b^Not applicable.

^c^Significant finding *P*=.01.

^d^CES-D Scale: Center for Epidemiological Studies–Depression Scale.

^e^ABS: Affect Balance Scale.

^f^No effect size due to no significant correlation between PTS and ABS-Negative Affect.

^g^Significant finding *P*=.05.

^h^SWLS: Satisfaction With Life Scale.

#### Congruent Construct Validity (Hypothesis 1.1): Correlations Between Psychologist in a Pocket and Depression Tests

PiaP’s construct, *depression symptoms*, was validated with 2 psychological tests of depression. Using PPMC, congruent construct validity was determined by correlating the participants’ (1) 1-week PTS with CES-D Scale scores and (2) 2-week PTS with BDI-II scores. These PiaP timeframes were considered as CES-D Scale instructs the respondents to recall depressive symptoms occurring for the week before testing, whereas BDI-II evaluates depressive symptoms for the previous 2 weeks before test administration. At 0.01 level of significance (2-tailed), results show significant low to moderate positive correlations between (1) PiaP and CES-D Scale (*r*=0.42, n=53, *P*=.002) and (2) PiaP and BDI-II (*r*=0.50, n=53, *P*<.001), respectively. Furthermore, Cohen *d* ’s ES values for 1-week PTS and CES-D Scale (*d*=0.42) and 2-week PTS and BDI-II (*d*=0.50) suggest a moderate to high practical significance, respectively.

#### Convergent Construct Validity (Hypothesis 1.2): Correlations Between Psychologist in a Pocket and Affect Balance Scale–Negative Affect

Although the correlations are positive, they are not significant. There is no significant correlation between the 2-week PTS and ABS–Negative Affect scores (*r*=0.19, n=53, *P*=.17). In addition, there is no significant correlation between the 1-week PTS and ABS–Negative Affect scores (*r*=0.25, n=53, *P*=.07). In addition, Cohen *d* ’s ES indices for both ABS–Negative Affect and (1) 1-week PTS (*d*=0.25) and (2) 2-week PTS (*d*=0.19) indicate low practical significance.

#### Divergent Construct Validity (Hypothesis 1.3): Correlations Between Psychologist in a Pocket with Affect Balance Scale–Positive Affect and Satisfaction With Life Scale

At 0.05 level of significance (2-tailed), a significant but negligible correlation exists between 1-week PTS and ABS–Positive Affect (*r*=−0.29, n=53, *P*=.04). A negative but nonsignificant relationship exists between 2-week PTS and ABS–Positive Affect (*r*=−0.20, n=53, *P*=.15). Cohen *d* ’s ES for both ABS–Negative Affect and (1) 1-week PTS (*d*=−0.29) and (2) 2-week PTS (*d*=−0.20) results are in the low practical significance range.

A significant but negligible correlation at 0.05 level of significance (2-tailed) was also obtained between SWLS and 1-week PTS (*r*=−0.29, n=53, *P*=.04), whereas there is a low positive significant correlation at 0.05 level of significance between SWLS and 2-week PTS (*r*=−0.32, n=53, *P*=.02). Cohen *d* ’s ES for SWLS and (1) 1-week PTS (*d*=−0.29) and (2) 2-week PTS (*d*=−0.32) scores are in the low to moderate practical significance range, respectively.

### Hypothesis 2: Concordance Analysis

**Figure 1:**
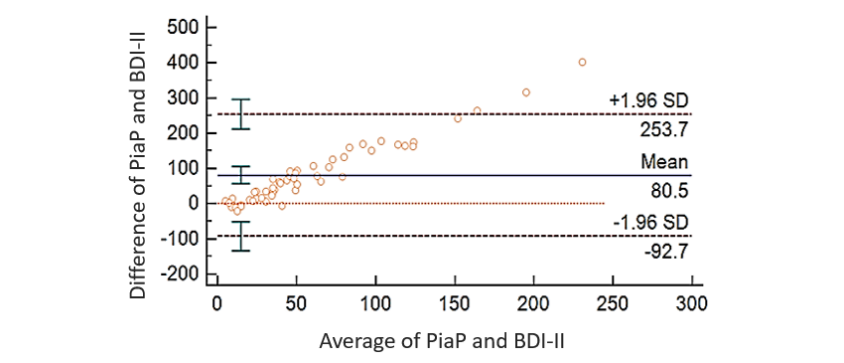
Bland-Altman plot analysis of Psychologist in a Pocket (PiaP) and Beck Depression Index-II (BDI-II).

MedCalc statistical software [50] was used to compute and to create the B-A plot. The concordance between the difference of PiaP and BDI-II scores and the average of PiaP and BDI-II scores is analyzed (Figure 1). Mean difference of raw scores is 80.50, which is within the CI of 56.1289 to 104.8522. Limits of agreement values are from −92.7 to 253.7. Upper confidence limit of 253.7 falls within the upper 95% CI limit (CIL; 211.8261 to 295.6209), whereas the lower confidence limit of −92.7 is within the range of the lower 95% CIL (−50.8449 to −134.6397). Out of 53 participants, only 3 were outliers.

## Discussion

### Primary Contribution

Together with our prior work on lexicon development and content validation [19], this work concludes the tripartite model of test construction on the PiaP. To the best of our knowledge, this is the first time a mobile mental health app has been validated according to the tripartite model of test construction.

Construct validity correlations show correlation with congruent construct, and the concordance analysis further indicates that the PiaP’s lexicon is able to reproduce standard test findings. In addition, PiaP is EMA-based and, therefore, does not rely on memory. Symptoms that are easily overlooked by psychological tests can be detected in a more timely manner. In addition, mobile phone–captured data might be more sensitive than paper-and-pencil–collected data [51]. Thus, PiaP can be an addition to the classical pen-and-paper tests and give a more detailed picture on mood changes.

Although the congruent correlation values of PiaP with the BDI-II and the CES-D Scale reflect that they measure the same construct, ES values quantify (1) the differences between PiaP with the 2 paper-and-pencil tests and (2) PiaP’s effectiveness to screen for depression symptoms via text analysis. Furthermore, this shows that mobile phones offer a platform where language can be studied and used to identify people with depression through their free texts and novel ways of communication. For PiaP users, this could mean a more feasible and comfortable way of reporting their symptoms, while providing a reliable, immediate, and more encompassing screening (and monitoring) of depression symptoms.

Although correlation for convergent and divergent constructs seem low, this is expected as high correlation should mostly occur for the congruent construct. Simply put, convergent and divergent constructs behave similar (or similar inverted) to the intended measure but not identical. Thus, no perfect correlation should be reached.

### General Remarks

More than 5000 observations or text inputs of depression-related words were made by PiaP during the 2-week test period. The resulting high SD values of PiaP scores indicate great variability in the number of responses between the participants. This variability is likely because of the nature of text inputs. Logging of text messages and text evaluations are based on free text inputs during daily usage without any specific prompts. This PiaP approach to depression detection is unlike structured psychological (depression) tests, wherein replies to target questions or stimuli require a specific kind of response. In addition, PiaP texts are captured in real time or close in time to experience, allowing for a steady and unlimited detection of numerous and varying mood changes.

The decrease in the number of depression text inputs from the participants (from 3154 inputs in week 1 to 2060 inputs in week 2) may be attributed to academic-related factors. In week 2 of data gathering, there was presumably lesser stress in the preparation of class requirements and exams before the Christmas break, whereas higher academic pressure in week 1 may have led to depression and anxiety [52] or perceived lack of achievement [53].

Low to moderate correlations between PiaP and the psychological tests utilized may be because of the restriction in the range of scores included in the sample. Restricted range occurs when the scores of 1 or both variables in a sample have a range of values that is less than the range of scores in the population [26], thus reducing the correlation found in a sample relative to the correlation that exists in the population. As only 53 participants successfully complied with the required 2-week PiaP run and the completion of psychological tests, this limited the range of scores available for analysis.

The large quantity of items or keywords in the PiaP lexicon may have contributed to the low or insignificant correlation results. This is not surprising as the psychometrics of word usage is in contrast with the typical test development such that compiled words in lexica are not normally distributed, have low base rates, and do not adhere to the traditional psychometric laws. Thus, standard reliability measures are not always appropriate in such a scenario [54].

### Hypothesis 1: Construct Validity Correlations

#### Congruent Construct Validation (Hypothesis 1.1)

The congruent construct validation attempts to determine whether the construct or attribute of the psychological approach in question correlates with a gold standard. Significant positive correlations with BDI-II and CES-D Scale imply that PiaP’s measure is compatible with the depressive symptoms measured in BDI-II and CES-D Scale. In addition, ES provides additional meaning to the results by providing more concrete and meaningful interpretations. In this study, ES ranged from medium to high, implying that depression signs are observable in their text inputs.

#### Convergent Construct Validation (Hypothesis 1.2)

Contrary to the study’s hypothesis, there is no significant correlation between depression and negative affect. This finding might be because of the fact that depression is a phenomenon with complex and varied features. In addition, the experience of depression might not be manifested through negative affect alone nor its absence demonstrated through positive affect or positive emotion. As Beck suggested in the cognitive theory of depression, negative thought processes and rumination, which are common and debilitating aspects of depression, should be the main focus of evaluation, as depression displays itself in negative thinking before it creates negative affect or mood [55].

#### Divergent Construct Validation (Hypothesis 1.3)

Divergent constructs of *positive affect* and *life satisfaction* were hypothesized to be inconsistent with the experience of depression.

*Positive affect* has a weak to negligible correlation. This suggests that, although positive affect has been shown to be low or absent in an individual experiencing depression, it is independent from negative affect, regardless of the intensity of affective experience [56]. Positive affect and negative affect are 2 broad mood factors which are salient in self-reported mood [33]. Having low levels of positive affect may not immediately point to negative affectivity but may be manifested as lethargy or fatigue. Among the participants, low levels of positive affect were consistently related only to depressive symptoms such as loss of pleasurable engagement.

*Life satisfaction* appears to be the stronger contrary attribute to depressive symptoms, as evidenced by the more stable and consistent negative correlation between PiaP and SWLS. Life satisfaction is a (negative) predictor of depression [57], second only to negative thoughts. Sample text inputs of research participants who obtained low scores in SWLS fall under the following PiaP categories: depressed mood, suicide, loss of interest, and fatigue.

### Hypothesis 2: Concordance Analysis (Bland-Altman plot and analysis)

Concordance analysis reveals that PiaP’s evaluation of depression symptoms via text or lexical analysis is comparable with the use of BDI-II, implying that PiaP is able to identify the presence of depressive symptoms similar to commonly used structured depression tests. It indicates that PiaP’s lexica are valid depression indicators as reflected in BDI-II. It likewise suggests that PiaP’s text analysis approach is able to reveal current psychological states, making it comparable with BDI-II’s appraisal of current symptoms of depression.

In addition, PiaP’s degree of agreement with BDI-II implies that it can support continued mental health appraisal, such as in an ongoing depression monitoring and screening of patients in between their appointments with doctors and/or therapy sessions.

### Limitations

One limitation of this work is the high dropout attrition rate. Despite having agreed to take part in both stages 2 and 3 of this study, a sizeable proportion of participants did not respond to follow-ups for stage 3. Although high attrition rates are avoided in traditional clinical trials, such a phenomenon is a naturally occurring and distinct feature of remote electronic health trials [58,59]. In addition, adherence to mental health care apps tend to be poor among individuals with mild to severe depression [60]. As a result of the high attrition rate, the final research group consisted only of 53 participants. This lower-than-expected sample size may undermine the study’s significant findings. However, the researchers applied the 3 approaches to external validation and, to strengthen the positive correlation results, added the B-A analysis particularly for the congruent construct validation. In addition, the medium-to-high ES values imply that the effectiveness of PiaP’s approach in identifying depression symptoms, as compared with paper-and-pencil tests, is consistent and obvious.

A second limitation of PiaP is the limitation to text input. Behavioral symptoms [61] or weight change and appetite disturbance [61] could be important in detecting a person with depression. The individual’s behavioral or motoric expressions of affect may not have been clearly detected as they are more difficult to verbalize. Hence, it is suggested that PiaP be validated with behavioral markers of depression such as movement and sleep patterns.

Finally, several results have either significant yet low correlation or no correlation. As previously mentioned, depression is a complex condition with cognitive, affective, and behavioral manifestations. As PiaP scoring relies on language usage, which tends to reflect the cognitive and affective elements of depression, the app is unable to screen for behavioral signs of depression, which cannot be expressed via text.

### Comparison With Prior Work

We compare our work with studies on mobile apps for depression in terms of (1) application of EMA, (2) lexicon development, and (3) construct validation.

First, PiaP, as it employs EMA, does its evaluation with a time stamp upon the exact occurrence of the symptoms using text analysis. Chung et al [62] designed a mobile app that recorded daily self-reported ratings for the Korean version of the Center for Epidemiologic Studies Depression Scale–Revised (K-CESD-R). Although the K-CESD-R Mobile app was completed by their 20 participants every day for 2 weeks to avoid recall bias, it still did not employ EMA real-time measurement unlike PiaP.

Second, PiaP considered the cultural expression of depression in text analysis in the creation of its English-Tagalog lexicon. This includes the mixed usage of Tagalog and English (Taglish), textolog (shortening of words), emoticons, and emojis, thus allowing for the recognition of “possible cultural variations in the expression of depressive symptoms via electronic data” [63,64] and providing a more nuanced screening. Compared with BinDhim et al [65], although they proved the feasibility of using a mobile app for depression screening by utilizing an app that was an electronic version of the Patient Health Questionnaire (PHQ)-9, they did not use text analysis.

Third, PiaP applied congruent construct validation to determine whether its construct of *depressive symptoms* corresponds to the depression construct of established psychological measures for depression. In Chung et al [62] and BinDhim et al [65] studies, each used only 1 test—K-CESD-R and PHQ-9, respectively as a basis for the electronic (mobile app) version. In the case of PiaP, aside from using CES-D Scale to determine construct validation of the PiaP lexicon, the researchers also used BDI-II, considered to be the gold standard in depression identification [66].

### Conclusions

A major point to consider from this study is that the language used in contemporary avenues (such as social media communication and mobile technology) serves as a channel for expressing depression-associated emotions while avoiding stigmatization, thereby making lexical data analysis an added dimension to depression-screening. Language—the use or choice of words—can express most depression symptoms that are better expressed in verbal behavior, specifically those that are more cognitive in nature. With social media and other forms of communication being incorporated in mobile phones, it becomes easier to express oneself for individuals who may be experiencing depression, as they prefer to spend more time online rather than have face-to-face interactions.

The study also alludes to the value of combining current technology with mental assessment. Mobile technology and, consequently, EMA should be maximized for a timely identification, screening, monitoring, and follow-up of individuals with depression and other mental health issues.

As an mHealth app for depression screening, PiaP provides several advantages. First, PiaP has proven both its internal [19] and external validities, thus satisfying the increasing need for the scientific testing of mHealth apps. With its reliance on EMA, PiaP provides prompt information regarding the user’s psychological state and eliminates or reduces errors and biases associated with interviews and self-reports of traditional mental health screening approaches, specifically in depression. Finally, PiaP’s lexical analysis of electronic data yields a layer of refinement to depression identification. With this leverage, PiaP can be used as an accessible and novel supplement and technological support to traditional approaches in depression screening and monitoring.

## Appendix

Multimedia Appendix 1 Psychologist in a Pocket (PiaP) research version opening message.

Multimedia Appendix 2 Psychologist in a Pocket (PiaP) set-up screen.

Multimedia Appendix 3 Presentation during the PAP 55th Annual Convention (20-22 Sept 2018, Manila, Phil).

## Abbreviations

ABS: Affect Balance Scale
B-A: Bland-Altman
BDI-II: Beck Depression Index-II
CES-D Scale: Center for Epidemiological Studies–Depression Scale
CIL: confidence interval limit
DSM-5: Diagnostic and Statistical Manual of Mental Disorders–5
EMA: ecological momentary assessment
ES: effect size
K-CESD-R: Korean version of the Center for Epidemiologic Studies Depression Scale–Revised
mHealth: mobile health
PHQ: Patient Health Questionnaire
PiaP: Psychologist in a Pocket
PPMC: Pearson product-moment correlation
PTS: PiaP total score
SWLS: Satisfaction With Life Scale
