# Supplementary material for: Validation of an mHealth App for Depression Screening and Monitoring (Psychologist in a Pocket): Correlational Study and Concurrence Analysis
Source: JMIR Mhealth Uhealth. 2019 Sep 16;7(9):e12051. doi: 10.2196/12051 (PMC6754681; doi:10.2196/12051)
Supplement: Multimedia Appendix 1 [file mhealth_v7i9e12051_app1.pdf]

6:26 26%

Android App Testing - Google Play

If you already have the **Psychologist in a Pocket** app installed on your device, you'll get an update with the testing version shortly. If you don't have the **Psychologist in a Pocket** app installed, download it on Google Play first, then you'll get an update with the testing version. It can take a few hours for the update to arrive.

Leave the testing program

You can leave the testing program at any time. If you leave the testing program and a public version of the app is available, you can switch to public version by uninstalling the testing version and installing the app again on Google Play.

Leave the program

You are a tester

Download the **Psychologist in a Pocket** app on Google Play

© 2015 Google - Google Play Terms of Service - Devices Terms of Sale - Privacy Policy - Developers - Artists
