# Supplementary figures and images for: Validation of an mHealth App for Depression Screening and Monitoring (Psychologist in a Pocket): Correlational Study and Concurrence Analysis
Source: JMIR Mhealth Uhealth. 2019 Sep 16;7(9):e12051. doi: 10.2196/12051 (PMC6754681; doi:10.2196/12051)

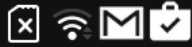

8:59

21%

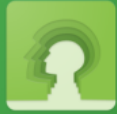

Psychologist in a Pocket

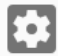

Set Up

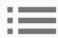

List Logs

Support the research!

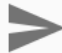

Share Encrypted Log Files

Supplement: Multimedia Appendix 2 [file mhealth_v7i9e12051_app2.pdf]
