# Supplementary material for: Validation of an mHealth App for Depression Screening and Monitoring (Psychologist in a Pocket): Correlational Study and Concurrence Analysis
Source: JMIR Mhealth Uhealth. 2019 Sep 16;7(9):e12051. doi: 10.2196/12051 (PMC6754681; doi:10.2196/12051)
Supplement: Multimedia Appendix 3 [file mhealth_v7i9e12051_app3.pdf]

ESTABLISHING MHEALTH APP INTEGRITY:

Validation of Psychologist in a Pocket in screening depression

PAULA FERRER CHENG, MA
UNIVERSITY OF SANTO TOMAS, MANILA
VIVECH SYSTEM SOLUTIONS INC
